# An Approach to Speed up Single-Frequency PPP Convergence with Quad-Constellation GNSS and GIM

**DOI:** 10.3390/s17061302

**Published:** 2017-06-06

**Authors:** Changsheng Cai, Yangzhao Gong, Yang Gao, Cuilin Kuang

**Affiliations:** 1 School of Geosciences and Info-Physics, Central South University, Changsha 410083, China; cscai@hotmail.com (C.C.); yangzhaogong@csu.edu.cn (Y.G.); cuilinkuang@163.com (C.K.); 2 School of Surveying and Urban Spatial Information, Beijing University of Civil Engineering and Architecture, Beijing 100044, China; 3 Department of Geomatics Engineering, University of Calgary, Calgary, AB T2N 1N4, Canada

**Keywords:** precise point positioning, single-frequency, convergence, GNSS

## Abstract

The single-frequency precise point positioning (PPP) technique has attracted increasing attention due to its high accuracy and low cost. However, a very long convergence time, normally a few hours, is required in order to achieve a positioning accuracy level of a few centimeters. In this study, an approach is proposed to accelerate the single-frequency PPP convergence by combining quad-constellation global navigation satellite system (GNSS) and global ionospheric map (GIM) data. In this proposed approach, the GPS, GLONASS, BeiDou, and Galileo observations are directly used in an uncombined observation model and as a result the ionospheric and hardware delay (IHD) can be estimated together as a single unknown parameter. The IHD values acquired from the GIM product and the multi-GNSS differential code bias (DCB) product are then utilized as pseudo-observables of the IHD parameter in the observation model. A time varying weight scheme has also been proposed for the pseudo-observables to gradually decrease its contribution to the position solutions during the convergence period. To evaluate the proposed approach, datasets from twelve Multi-GNSS Experiment (MGEX) stations on seven consecutive days are processed and analyzed. The numerical results indicate that the single-frequency PPP with quad-constellation GNSS and GIM data are able to reduce the convergence time by 56%, 47%, 41% in the east, north, and up directions compared to the GPS-only single-frequency PPP.

## 1. Introduction

Precise point positioning (PPP) is a single-receiver absolute positioning technique that uses un-differenced pseudorange and carrier phase observations along with precise satellite orbit and clock products [1]. How to reduce the PPP convergence time has become a key issue for further improving PPP performance [2,3,4,5]. The PPP technique conventionally uses dual-frequency observations to remove the first-order ionospheric delay errors. Given that the majority of mass-market global navigation satellite system (GNSS) users are operating single-frequency receivers, the dual-frequency PPP technique has been extended to single-frequency PPP [6,7,8]. In the dual-frequency PPP, the ionospheric delay error can be eliminated by forming an ionosphere-free linear combination between dual-frequency observations. However, in the single-frequency PPP, the ionospheric delay becomes a major error source to be mitigated.

Two different methods have been proposed for ionospheric delay error mitigation in the single-frequency PPP. One is to apply ionospheric models, such as the global ionospheric map (GIM), to mitigate the ionospheric effect [6,9,10], but this method can only achieve decimeter-level positioning accuracy due to the accuracy limitation of the ionospheric model [10]. For instance, the GIM model only has an accuracy of 2–8 TECU (total electron content unit), which is equivalent to a ranging error of 0.32–1.28 m for the GPS L1 frequency signal. The second method is to form an ionosphere-free combined observable between the code and carrier phase observations on a single frequency, which is known as GRoup And PHase Ionospheric Correction (GRAPHIC) [11,12,13,14]. Based on this GRAPHIC method, an accuracy of several centimeters is achievable, but a long convergence time of over two hours is required [7]. The long convergence time is attributed to two aspects. On one hand, the GRAPHIC combined observables are dominated by the noise of the code measurements. On the other hand, the GRAPHIC combination from single-frequency code and phase observations produces a rank-deficient mathematical problem [15]. In summary, both methods suffer major limitations where the first method converges more quickly, but can only achieve decimeter-level positioning accuracy while the second method can achieve centimeter-level positioning accuracy, but requires a very long convergence time. 

In this study, an approach to combine both methods is proposed, which can speed up the convergence and achieve high positioning accuracy at the same time. Further, instead of applying the GRAPHIC combination, the ionospheric and hardware delays (IHD) are estimated together as a single unknown parameter. The IHD values obtained from the GIM product and the multi-GNSS differential code bias (DCB) product are used as pseudo-observables to estimate the IHD parameter. A time varying weight scheme has also been proposed for the pseudo-observables to gradually decrease their contribution to the position solutions. Since the multi-GNSS signals have been widely demonstrated to be capable of improving the PPP performance [14,16,17,18,19],the quad-constellation measurements will be utilized with the proposed approach to further accelerate the convergence. Presently, GPS and GLONASS systems are providing global navigation services. The BeiDou system (BDS) provides a regional navigation service and the Galileo system offers an initial global navigation service. 

The rest of the paper is organized as follows: Firstly, a single-frequency PPP approach of combining quad-constellation GNSS and GIM data is presented. Secondly, the GIM products and the multi-GNSS DCB Products from different agencies are compared and evaluated by GNSS measurements. Finally, the converging performance of the proposed single-frequency PPP approach is assessed. 

## 2. Approach of Single-Frequency PPP with Quad-Constellation GNSS and GIM

The code and carrier-phase observations on a single frequency can be expressed as:

(1) $$P^{g}=\rho^{g}+cdT-cdt^{g}+d_{orb}^{g}+d_{trop}^{g}\;+d_{ion}^{g}+b^{g}+\varepsilon_{P}^{g}$$

(2) $$\Phi^{g}=\rho^{g}+cdT-cdt^{g}+d_{orb}^{g}+d_{trop}^{g}\;-d_{ion}^{g}+B^{g}+\varepsilon_{\Phi }^{g}$$

where the superscript *g* represents a GPS satellite, *P* is the measured pseudorange in meters, Φ is the measured carrier phase in meters, *ρ* is the geometric range in meters, *c* is the speed of light in meters per second, *dT* is the receiver clock offset in seconds, *dt* is the satellite clock offset in seconds, *d_orb_* is the satellite orbit error in meters, *d_trop_* is the tropospheric delay in meters, *d_ion_* is the ionospheric delay in meters, *B* is a bias term in meters, including the phase ambiguity and the phase hardware delay bias, *b* is the code hardware delay bias at satellite and receiver ends in meters, and *ɛ_P_* and *ɛ*_Φ_ are the code and phase observation noises including multipath errors in meters, respectively. In the PPP parameter estimation, the code hardware delay at the receiver end will be absorbed by the receiver clock offset and, thus, only the code hardware delay bias at the satellite end needs to be considered. Since the precise satellite clock corrections to be used contain the ionosphere-free linear combinations of the satellite code hardware delays on two frequencies, single-frequency users must apply the satellite DCB to obtain the proper code hardware delay on the chosen frequency [18].

Since GPS, GLONASS, BeiDou, and Galileo systems have their respective time systems, a receiver clock parameter with respect to their own time scale should be introduced for each constellation although there is a unique physical receiver clock for the positioning model [17]. Alternatively, the system time difference parameters with respect to a reference time scale may be introduced [3]. If the GPS time scale is chosen as a reference, the GLONASS, BDS, and Galileo observation models can be depicted by:

(3) $$P^{j}=\rho^{j}+cdT+cdT_{sys}^{j,g}-cdt^{j}+d_{orb}^{j}+d_{trop}^{j}\;+d_{ion}^{j}+b^{j}+\varepsilon_{P}^{j}$$

(4) $$\Phi^{j}=\rho^{j}+cdT+cdT_{sys}^{j,g}-cdt^{j}+d_{orb}^{j}+d_{trop}^{j}\;-d_{ion}^{j}+B^{j}+\varepsilon_{\Phi }^{j}$$

where *j* represents GLONASS, BeiDou, and Galileo satellites, *dT_sys_* is the system time difference with respect to the GPS time scale.

In Equations (1)–(4), the ionospheric delay and the code hardware delay are lumped together and thus they can be estimated as a single unknown parameter, namely *d*_IHD_ = *d*_ion_ + *b*. If there is no restriction to be imposed on the unknown parameter, the uncombined single-frequency PPP model is practically equal to the GRAPHIC ionosphere-free model. In order to reduce the PPP convergence time, the ionospheric delay and code hardware delay derived from the GIM product and multi-GNSS DCB product are applied as pseudo-observables to constrain the *d*_IHD_ parameter, as seen below.

(5) $$I_{slant}+b_{hd}=d_{IHD}$$

(6) $$I_{slant}=40.28\times \frac{m\cdot VTEC_{GIM}}{f^{2}}$$

(7) $$m={\left[1-\frac{\sin^{2}z}{{\left(1+H_{ion}/R\right)}^{2}}\right]}^{-1/2}$$

(8) $$b_{hd}=\frac{{f_{2}}^{2}}{{f_{1}}^{2}-{f_{2}}^{2}}DCB_{P_{1}P_{2}}$$

where *I_slant_* is the pseudo-observable of the ionospheric delay, which can be derived by Equation (6). *b_hd_* is the pseudo-observable of the code hardware delay, which can be acquired through DCB by Equation (8) [18]. In Equation (6), *m* is the ionospheric mapping function as expressed in Equation (7), *f* is the carrier frequency, *VTEC*_GIM_ is the interpolated total electron content (TEC) value along the zenith direction from the TEC map data [20]. In Equation (7), *z* is the zenith angle of the line of sight at the receiver, *R* is the average radius of the Earth, and *H*_ion_ is the height of the assumed ionosphere single layer, which is 450 km for most of the GIM products.

The variance of the code and carrier phase observations can be expressed as the function of the initial variance $\sigma_{0}^{2}$ and the satellite elevation angle *E*:

(9) $$\sigma^{2}=\sigma_{0}^{2}/{\left(\sin E\right)}^{2}$$

For the pseudo-observables of the IHD, their variance can be expressed as:

(10) $$\sigma_{IB}^{2}=\sigma_{IB\_t}^{2}\cdot m^{2}$$

where $\sigma_{IB\_t}^{2}$ is the IHD pseudo-observable variance in the zenith direction. Since the GIM products only have an equivalent ranging accuracy of 0.32–1.28 m, the accuracy of the IHD pseudo-observables derived from the GIM and DCB products would be lower than 0.32 m. To account for this limitation, a time varying weight scheme is proposed for the pseudo-observables. The idea behind the scheme is that the pseudo-observables are assigned a larger weight at the beginning of data processing for the purpose of faster convergence but with gradually decreased weights during the convergence period for the purpose of better positioning accuracy. A Kalman filter algorithm is normally applied in the PPP processing. The $\sigma_{IB\_t}^{2}$ in Equation (10) in this case can be expressed as a function of the filtered time in the Kalman filter:

(11) $$\sigma_{IB\_t}^{2}=\sigma_{IB\_0}^{2}+\alpha \cdot \Delta t$$

where $\sigma_{IB\_0}^{2}$ is the initial variance of the pseudo-observables, *α* is the variance varying rate with time, Δt is the filtered time in the Kalman filter processing.

Equations (1)–(5) are the observation equations of the single-frequency PPP model with quad-constellation GNSS and GIM. The precise satellite orbit and clock corrections from international GNSS service (IGS) are applied to mitigate the satellite orbit and clock errors. The mitigation of other error sources may refer to [1]. The carrier phase cycle slips are detected and repaired using a forward and backward moving window averaging algorithm and a second-order, time-difference phase ionospheric residual algorithm [21]. The unknown parameters of the proposed single-frequency PPP model include the three-dimensional position coordinates, the receiver clock offset, the system time differences, the zenith tropospheric delays, the ambiguities and the IHD. The static position coordinate and ambiguity parameters are considered as constants while the other parameters are modeled as random walk processes in the Kalman filter processing.

## 3. GIM and DCB Products

The IGS ionosphere working group has started to produce the ionosphere vertical TEC maps since June 1998. Four IGS ionosphere associate analysis centers (IAACs) have released global TEC maps in agreement with the IONosphere map EXchange (IONEX) format [20], also referred as so-called GIM. The four IAACs are CODE (Center for Orbit Determination in Europe, Switzerland), ESOC (European Space Operations Center, Germany), JPL (Jet Propulsion Laboratory, USA) and UPC (Technical University of Catalonia, Spain). As a byproduct of these TEC maps, DCB values from both satellite and tracking receiver ends are estimated and provided as well. Each IAAC computes the rapid and final TEC maps independently using different approaches. The GIM products from CODE and JPL are combined to form IGS GIM products.

GIM contains TEC values along the zenith direction, i.e., vertical TEC (VTEC), in a globally-distributed grid. In latitude, grid points range from +87.5° to −87.5° with a spatial resolution of 2.5°. In longitude, grid points are arranged from −180° to +180° with a resolution of 5°. The TEC map is updated at an interval of one or two hours. According to the IONEX format [20], the GIM is provided in an Earth-centered-Earth-fixed (ECEF) reference frame in spherical coordinates rather than the geographical coordinates.

The multi-GNSS DCB can be determined by using a priori ionospheric models or jointly estimating the DCB bias and ionosphere delay [22]. Currently, two analysis centers, namely, Deutsches Zentrum für Luft- und Raumfahrt (DLR) and the Chinese Academy of Sciences (CAS), provide the multi-GNSS DCB products, covering GPS, GLONASS, BDS and Galileo multi-frequency signals. The two DCB products exhibit a consistency of about 0.2 ns and 0.4 ns for Galileo and BDS, respectively [23]. In comparison with GPS and GLONASS DCB products generated by CODE, the multi-GNSS products exhibit RMS (root mean square) differences of approximately 0.2 ns and 0.6 ns, respectively. Monthly repeatabilities of the multi-GNSS DCBs are at the level of 0.1 ns for GPS and Galileo and 0.2 ns for BDS and GLONASS [23].

**Figure 1:**
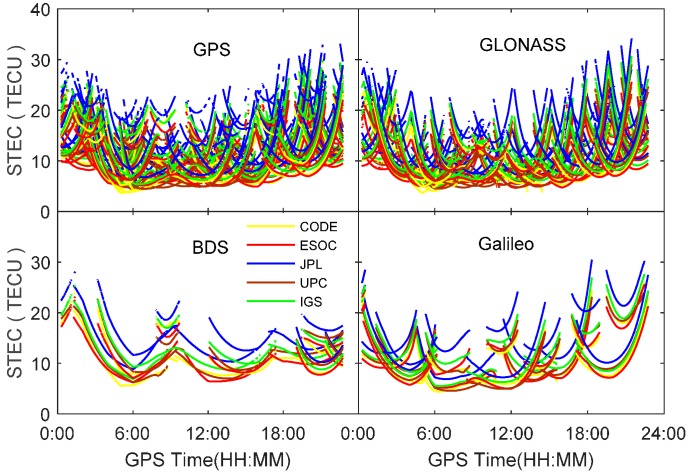
Slant TEC derived from different agencies at station PFRR on 4 September 2016.

**Figure 2:**
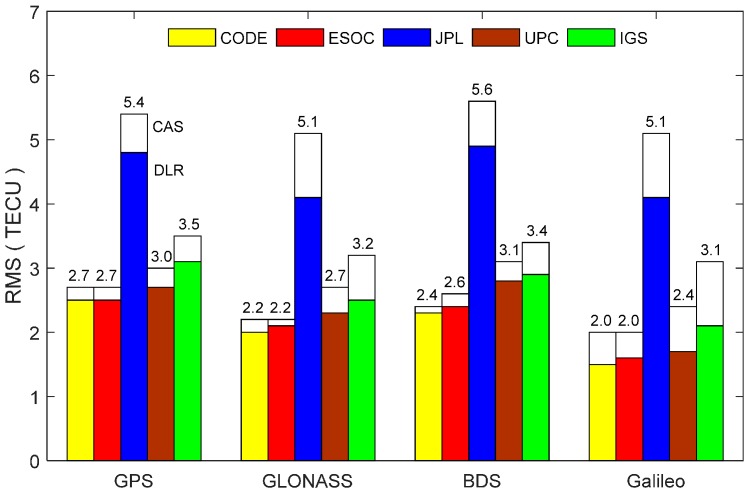
RMS statistical values of slant TEC derived from different GIM products based on the CAS (white bar) and DLR (color bar) multi-GNSS DCB corrections.

In order to compare the GIM products generated from different agencies, the TEC along the line of sight at each ionospheric pierce point of satellite signals are computed using datasets at station PFRR on 4 September 2016 by applying Equations (6) and (7). Figure 1 shows the derived slant TEC (STEC) for all visible satellites from different constellations. It can be seen that there exist obvious offsets for GIM products provided by different agencies, although their varying trend is quite consistent. The largest TEC discrepancy is up to 10.5 TECU for GIM products provided by JPL and ESOC. In order to assess the GIM products, the dual-frequency observations from four constellations are utilized to derive the line-of-sight TEC of each satellite as references. The DCBs at both satellite and receiver ends are corrected using the multi-GNSS DCB products to obtain absolute TEC values. For further comparison, the CAS and DLR quad-constellation DCB products are employed, respectively. Figure 2 shows the RMS statistical values of the slant TEC derived from different agencies’ GIM and DCB products. As seen from the two figures, the CODE GIM products achieve the highest accuracy in terms of the RMS values. Comparing the white bars and the color bars for each constellation, it is explicit that the DLR DCB products slightly outperforms the CAS DCB products. Based on this analysis, the CODE GIM products and the DLR multi-GNSS DCB products are, therefore, adopted in our study.

## 4. Single-Frequency PPP Processing Results and Analysis

### 4.1. Data Description

**Figure 3:**
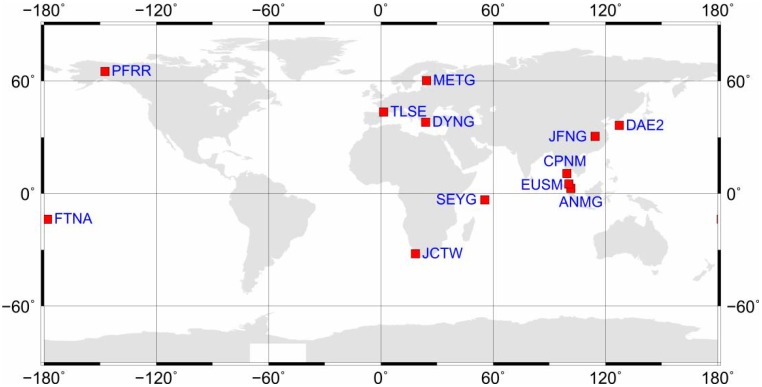
Geographical distribution of 12 MGEX stations.

Quad-constellation observation datasets collected from 12 globally-distributed MGEX stations, as well as the CODE GIM products and the DLR multi-GNSS DCB products on 4–10 September 2016 are used to assess the performance of the proposed single-frequency PPP approach. The distribution of stations is shown in Figure 3. All observations have a sampling rate of 30 s. In the PPP processing, the satellite elevation mask angle is set to 10°. The quad-constellation mixed satellite orbit and clock products provided by CODE are used at sampling intervals of 15 min and 5 min, respectively. Since the precise coordinates of most stations are not available, their coordinate values are computed at the precise millimeter level through an Online Positioning User Service (OPUS) that is developed by the United States’ National Geodetic Survey [24]. The OPUS processing results also refer to “IGS08”, which is consistent to the coordinate reference of the PPP solutions.

The spectral density values for the tropospheric zenith wet delay, the receiver clock offset, the system time difference and the IHD parameters are empirically set to 10^−9^, 10^5^, 10^−7^, and 10^2^ m^2^/s, respectively. Both GPS and GLONASS code and phase observation precisions are set to 0.3 m and 0.002 m, respectively. Their IHD pseudo-observable variance is initially set to 0.025 m^2^ and then gradually increases at a speed rate of 0.04 m^2^/min. Given that the BeiDou and Galileo constellations are still incomplete and their satellite orbit and clock corrections are at a relatively lower accuracy [25,26], their observations are down-weighted by a factor of four.

### 4.2. Processing Results and Analysis

In order to evaluate the proposed single-frequency PPP approach, the datasets at FTNA and SEYG stations on 5 September 2016 are processed in five different scenarios i.e., GPS, GPS/GLONASS, GPS/GLONASS/BDS, GPS/GLONASS/BDS/Galileo, and GPS/GLONASS/BDS/Galileo plus GIM. 

**Figure 4:**
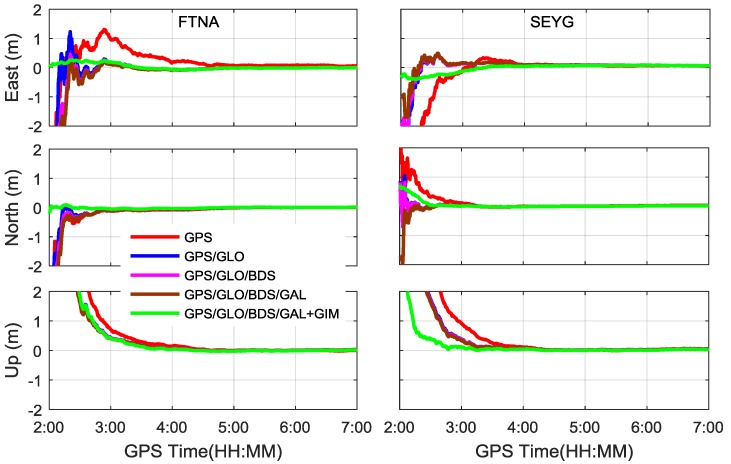
Single-frequency PPP errors for different processing scenarios using datasets at FTNA and SEYG stations on 5 September 2016.

**Figure 5:**
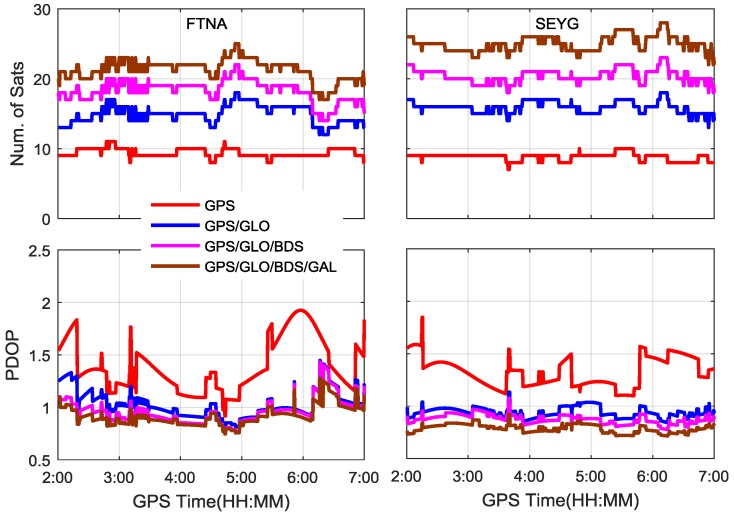
Number of satellites and PDOP for different PPP processing scenarios using datasets at FTNA and SEYG stations on 5 September 2016.

Figure 4 shows the PPP positioning errors based on the five processing scenarios. It is obvious that the GPS-only PPP solutions need a longer time to converge to stable values than the other constellation combinations. By combination of GPS with GLONASS, the GPS/GLONASS PPP has achieved significantly better convergence performance. As the BeiDou and Galileo observations have been assigned smaller weights than the GPS and GLONASS observations, the triple-constellation and quad-constellation PPP results are quite similar to the GPS/GLONASS dual-constellation PPP solutions, as seen from the blue, pink, and brown curves. After the GIM data is introduced, the quad-constellation PPP convergence performance is considerably improved, especially in the horizontal directions. Figure 5 shows the number of satellites and PDOP (position dilution of precision) for each processing scenario. Similar to the positioning error curves, the GPS/GLONASS dual constellations significantly improve the PDOP over the GPS-only single constellation. With integration to BDS and Galileo, the further improvement of PDOP is very limited, which also explains why the convergence performance cannot be notably enhanced with inclusion of BDS and Galileo.

Table 1 provides the RMS statistics in the east, north and up coordinate components, as well as RMS for the three-dimensional (3-D) position to demonstrate a positioning accuracy in five-hour sessions. The RMS computations are based on the position solution errors within the last 15 min. It can be seen that the final positioning accuracy reaches a few centimeters and is quite close for different scenarios. 

### 4.3. Convergence Performance Assessment

**Figure 6:**
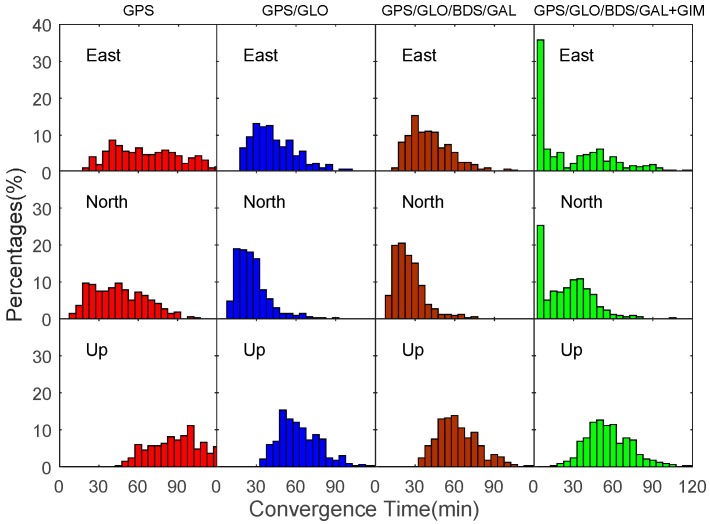
Distribution of convergence time for GPS-only, GPS/GLONASS, GPS/GLONASS/BDS/Galileo, and GPS/GLONASS/BDS/Galileo plus GIM single-frequency PPP using datasets at 12 MGEX stations over seven days.

In order to assess the convergence time of our proposed single-frequency PPP approach, the convergence time of GPS-only PPP, GPS/GLONASS PPP, and GPS/GLONASS/BDS/Galileo PPP are compared with the quad-constellation GNSS plus GIM PPP using datasets collected at twelve MGEX stations over seven consecutive days during 4–10 September 2016. In this study, the position filter is considered to have converged when the absolute values of the positioning errors in the horizontal directions reach 0.3 m and keep within 0.3 m. Given that the PPP errors in the height component are relatively larger than its horizontal components, the convergence criterion in the vertical directions is enlarged to 0.5 m. The convergence time is the period from the first epoch to the converged epoch. For each day, datasets are divided into five-hour sessions to ensure convergence of position solutions so each day includes four complete sessions. Datasets in each session are processed independently. As the DYNG dataset on 5 September 2016 is missed, a total of 332 sets of results are obtained to derive a statistical estimate on the convergence time as well as the positioning accuracy. The distribution of the 332 sets of convergence time in the east, north and up components is plotted in unit of minutes in Figure 6. It is visible that the GPS/GLONASS combination distinctly reduces the convergence time over the GPS-only PPP. This convergence time is further reduced in the quad-constellation PPP, but to a slight degree. In conjunction with the GIM data, the convergence time is significantly decreased especially in the horizontal directions. Using our proposed single-frequency PPP approach, the horizontal position solutions accounting for over 25% can converge within 5 min. The reason why the convergence performance can be improved after the GIM data is introduced is because the correlations among parameters of the IHD, receiver clock offset and ambiguities are decreased. The vertical direction is more sensitive to the ionospheric errors. As a result, the improvement in the vertical direction is less significant than the horizontal directions due to the limited accuracy of the GIM data.

Table 2 lists the convergence time of single-frequency PPP for different processing scenarios. The percentages in the brackets refer to the improvement over the GPS-only case. The average convergence time for the GPS-only case is 68 min, 43 min, and 93 min in the east, north, and up directions. With the integration of GPS and GLONASS, the convergence time is reduced by 38%, 42%, and 34% in the three directions, respectively. Using our proposed single-frequency PPP approach, the convergence time is further improved down to 30 min, 23 min, and 55 min in the east, north, and up directions. Compared to the GPS-only case, the improvement rate reaches 56%, 47%, and 41% in the three directions, respectively. It is noted that the east coordinate component needs longer convergence time than the north coordinate component for the reason of the satellite constellation configuration [17]. As a result, the improvement of the convergence time in the east direction is more significant after adding GIM data.

**Figure 7:**
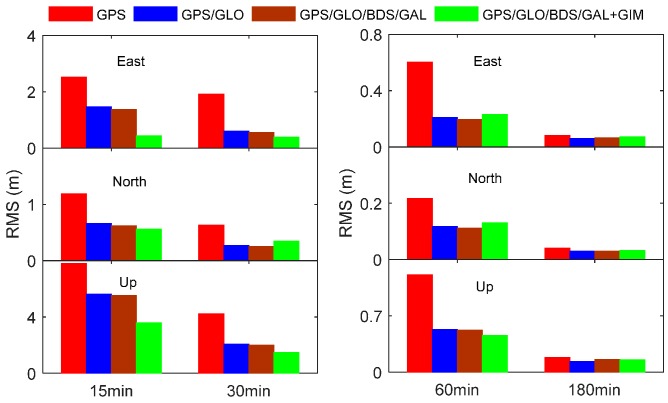
RMS statistics of positioning errors for different single-frequency PPP processing scenarios at the end of 15 min, 30 min, 60 min, and 180 min.

During the converging procedure of the PPP position filter, the size of position errors over time can also reflect the converging speed of the position filter. Figure 7 illustrates the RMS statistical values of position solutions for all stations and sessions at the end of 15 min, 30 min, 60 min, and 180 min sessions, respectively. From the statistical results of 15 min, it is noted that the positioning errors sharply decrease after introducing GIM data at the beginning of the filter processing. Undoubtedly, the GIM data plays a crucial role in accelerating the position filter convergence in the early processing stage. Progressively, the contribution of GIM data is lowered since the IHD pseudo-observables have been assigned smaller and smaller weights over time because of its limited accuracy. As a result, the GIM data quality has not caused significant effect on the converged position accuracy, as seen from the statistical results after 60 min and 180 min. In terms of the RMS statistics of positioning errors at the end of 180 min, our proposed single-frequency PPP approach can achieve a position accuracy of 0.072 m, 0.032 m, and 0.153 m in the east, north, and up directions, respectively. Overall, the proposed approach can not only speed up the filter convergence, but also achieve the high accuracy of position solutions. 

## 5. Conclusions

A significant challenge for the single-frequency PPP to achieve high-accuracy position solutions is its long convergence time of up to a few hours. In this study, an approach is proposed to speed up the single-frequency PPP convergence by combing the quad-constellation GNSS and GIM data. In this approach, the ionospheric delay and code hardware delay are estimated together as a single unknown parameter in an un-combined PPP observation model. The slant ionospheric delay and code hardware delay acquired from the GIM and multi-GNSS DCB products are introduced to the observation model as pseudo-observables with a time varying weight. 

Datasets collected at twelve MGEX stations on seven consecutive days are exploited to evaluate the proposed single-frequency PPP approach. Different agencies’ GIM products and multi-GNSS DCB products are compared and evaluated using GNSS measurements. Results indicate that the CODE GIM products and the DLR DCB products exhibit higher accuracy. A total of 332 datasets are utilized to assess the convergence performance of our proposed approach. Numerical results indicate that the quad-constellation GNSS plus GIM PPP reduces the convergence time by 56%, 47%, and 41% in the east, north, and up directions over the GPS-only PPP, respectively. With the inclusion of the GIM data, the single-frequency PPP can considerably reduce the positioning errors in the early stage of the filter processing. By using a time varying weight scheme, the final positioning accuracy is not affected by the GIM data quality. As a result, the horizontal position accuracy of several centimeters can be achieved using the single-frequency measurements collected by geodetic-type receivers.

## Figures and Tables

**Table 1:** RMS statistics of single-frequency PPP coordinate deviations for five different processing scenarios (m).

		GPS	GPS/GLO	GPS/GLO/BDS	GPS/GLO/BDS/GAL	GPS/GLO/BDS/GAL+GIM
FTNA	East	0.053	0.001	0.001	0.002	0.009
	North	0.005	0.006	0.006	0.008	0.010
	Up	0.008	0.027	0.028	0.025	0.016
	3-D	0.054	0.027	0.029	0.026	0.020
SEYG	East	0.065	0.059	0.062	0.058	0.067
	North	0.030	0.017	0.017	0.017	0.014
	Up	0.037	0.060	0.060	0.051	0.036
	3-D	0.081	0.086	0.087	0.079	0.078

**Table 2:** Average convergence time for single-frequency PPP (min).

		GPS	GPS/GLO	GPS/GLO/BDS/GAL	GPS/GLO/BDS/GAL+GIM
Convergence Time	East	68	42 (38%)	41 (40%)	30 (56%)
	North	43	25 (42%)	23 (47%)	23 (47%)
	Up	93	61 (34%)	61 (34%)	55 (41%)
